# Long non-coding and coding RNAs characterization in Peripheral Blood Mononuclear Cells and Spinal Cord from Amyotrophic Lateral Sclerosis patients

**DOI:** 10.1038/s41598-018-20679-5

**Published:** 2018-02-05

**Authors:** Stella Gagliardi, Susanna Zucca, Cecilia Pandini, Luca Diamanti, Matteo Bordoni, Daisy Sproviero, Maddalena Arigoni, Martina Olivero, Orietta Pansarasa, Mauro Ceroni, Raffaele Calogero, Cristina Cereda

**Affiliations:** 1 Genomic and post-Genomic Center, IRCCS Mondino Foundation, Pavia, Italy; 2 Department of Biology and Biotechnology “L. Spallanzani”, University of Pavia, Pavia, Italy; 3 Department of Brain and Behavioral Sciences, University of Pavia, Pavia, Italy; 4 General Neurology, IRCCS Mondino Foundation, Pavia, Italy; 5 Department of Molecular Biotechnology and Health Sciences, Bioinformatics and Genomics Unit, University of Torino, Torino, Italy; 6 Department of Oncology, University of Torino School of Medicine, Candiolo, Italy

## Abstract

Alteration in RNA metabolism, concerning both coding and long non-coding RNAs (lncRNAs), may play an important role in Amyotrophic Lateral Sclerosis (ALS) pathogenesis. In this work, we performed a whole transcriptome RNA-seq analysis to investigate the regulation of non-coding and coding RNAs in Sporadic ALS patients (SALS), mutated ALS patients (*FUS, TARDBP* and *SOD1*) and matched controls in Peripheral Blood Mononuclear Cells (PBMC). Selected transcripts were validated in spinal cord tissues. A total of 293 differentially expressed (DE) lncRNAs was found in SALS patients, whereas a limited amount of lncRNAs was deregulated in mutated patients. A total of 87 mRNAs was differentially expressed in SALS patients; affected genes showed an association with transcription regulation, immunity and apoptosis pathways. Taken together our data highlighted the importance of extending the knowledge on transcriptomic molecular alterations and on the significance of regulatory lncRNAs classes in the understanding of ALS disease. Our data brought the light on the importance of lncRNAs and mRNAs regulation in central and peripheral systems, offering starting points for new investigations about pathogenic mechanism involved in ALS disease.

## Introduction

There is mounting evidence that altered RNA metabolism, both involving coding and non-coding RNAs (ncRNAs), plays an important role in Amyotrophic Lateral Sclerosis (ALS) pathogenesis. ALS is an adult-onset, progressive and fatal neurodegenerative disease, caused by the selective loss of both upper and lower motor neurons in the cerebral cortex, brainstem and spinal cord. The pathogenesis of the disease is still unknown. Alterations of transcription regulation may represent key events in ALS pathogenesis, supported by mapping of genetic mutations in *TDP-43* and *FUS/TLS* genes coding for DNA/RNA binding proteins involved in transcription and RNA processing^1^. The same notion is strengthened by the observation that SOD1, Alsin and ANG are involved in transcription and processing of both coding and non-coding RNAs^2^ and by the finding that mutant SOD1 induces alternative splicing deregulation^1^.

About RNAs, there is a clear evidence of the importance of non-coding RNAs in central nervous system (CNS) functions and their involvement in neurodegenerative diseases such as Parkinson’s disease, Alzheimer’s disease and ALS. In addition, while microRNA involvement in neurodegenerative disorders has been the subject of intense research^3,4^, the recently revealed class of long non-coding RNAs (lncRNAs) is at the beginning of its characterization. LncRNAs are RNA transcripts greater than 200 nucleotides that lack an open reading frame and therefore do not encode proteins. While coding genes are widely annotated, high-quality catalogues of lncRNAs and tissue-specific expression data are recently being constructed. Recent efforts are directed to characterize this, largely unexplored, functional component of the genome. GENCODE consortium^5^, within the framework of ENCODE project, with a mixed approach based on manual annotation and ENSEMBL based annotation^6^, has categorized lncRNAs in different biotypes, based on their location with respect to protein-coding genes^7^: i) antisense (AS) RNAs have transcripts that overlap the genomic span of a protein-coding locus on the opposite strand, or published evidence of antisense regulation of a coding gene; ii) lincRNAs are intergenic non-coding RNAs; iii) sense overlapping RNAs contain a coding gene in an intron on the same strand; iv) sense intronic RNAs are present in introns of a coding gene and do not overlap any exon; v) processed transcript do not contain an ORF and cannot be added to previous biotypes.

LncRNAs are mostly related to possible regulator on biogenesis, cellular cycle and differentiation^8^, and are involved in nervous system and neurological diseases^9,10^. LncRNAs can act both as epigenetic regulators of target genes and as components of an extensive, unexplored network of interacting RNAs involving miRNAs and mRNAs. The literature has put in light the role of lncRNAs in both microsatellite expansion, i.e. Huntington disease^11^ and neurodegenerative diseases^9,12,13^ (Alzheimer’s and Parkinson’s Disease). Moreover, in the case of ALS neurodegenerative disease, few data are available about lncRNAs^14–16^. So far, it has been described that Nuclear-Enriched Abundant Transcript1 (*NEAT1*) generates two types of long non-coding RNAs. The named *NEAT1_2* lncRNA interacts with paraspeckle formation in spinal motor neurons of ALS patients^14^. It was indeed demonstrated that *NEAT1_2* lncRNA is up-regulated in spinal cord during the early stage of ALS pathogenesis compared to healthy controls^14^. Moreover, in patients affected by Frontotemporal Lobar Degeneration^17^, TDP-43 and FUS proteins bind to and regulate the expression of different lncRNAs^1^, although the mechanistic connection between lncRNAs and coding RNAs is not explained, yet.

The involvement of coding RNA deregulation on ALS has been demonstrated^18,19^ and different papers in the past years, have described this aspect, considering involved pathways or ALS mutated genes, such as SOD1^18^, TARDBP^20^. To reinforce the thesis of a whole coding RNA deregulation, recent studies, based on deep sequencing of coding RNAs both in monocytes^21^ and in spinal cord^22^ from ALS patients and matched controls, have been published.

In this paper, we present a whole transcriptome profiling of both long non-coding and coding RNAs in Peripheral Blood Mononuclear Cells of Sporadic ALS (SALS) patients and matched controls. LncRNAs data have been validated in spinal cord, as main involved tissues in ALS and the study was extended to a little group of patients with mutation in genes associated to ALS, i.e. *FUS*, *SOD1* and *TARDBP*. Furthermore, we have investigated the presence of co-expression networks between coding and lncRNAs.

## Results

### Deep sequencing lncRNAs expression profiles in PBMC samples of ALS patients and healthy subjects

We detected differentially expressed lncRNAs (DE lncRNAs) in PBMCs in five groups of subjects: sporadic patients (SALS), FUS, TARDBP and SOD1 mutated patients and healthy controls. In SALS patients, 293 DE lncRNAs were identified: 62.5% (183 out of 293) were up-regulated (Table 1). 62.8% (184 out of 293) were reported as antisense, 27.7% (81 out of 293) as lincRNAs, while the remaining 9.5% (28 out of 293) were classified as processed transcripts or intronic sense RNAs (Table 2). Only 23 out of the 293 DE lncRNAs were described as “known” transcripts, (i.e.: represented in the HUGO Gene Nomenclature Committee database and RefSeq), while remaining 270 transcripts were reported as “novel” (i.e.: transcripts containing four or more exons and/or supported by at least one mRNA/cDNA or three ESTs but not still present in common databases) (Table 2).

**Table 1:** Statistically significant differentially expressed mRNAs and lncRNAS number in PBMC from ALS patients, in terms of up-regulated transcripts, down-regulated transcripts and total. Counts are reported for sporadic ALS patients (SALS) and for mutated patients (FUS, TARDPB and SOD1).

	SALS		FUS		TARDB		SOD1	
	mRNAs	lncRNAs	mRNAs	lncRNAs	mRNAs	lncRNAs	mRNAs	lncRNAs
UP-regulated	57	183	35	16	10	6	14	0
DOWN-regulated	30	110	87	5	20	9	4	2
total	87	293	122	21	30	15	18	2

Transcripts were considered as differentially expressed when |log_2_(disease sample/healthy control)| ≥ 1 and a FDR ≤ 0.1.

**Table 2:** Classification of differentially expressed lncRNAs for biotypes and status according to GENCODE annotation for sporadic ALS patients (SALS) and for mutated patients (FUS, TARDPB and SOD1).

	**SALS**	**FUS**	**TARDBP**	**SOD1**
**gene biotype**				
antisense	184	11	7	2
lincRNA	81	6	6	0
processed transcript	20	2	1	0
sense intronic	8	2	1	0
**gene status**				
known	23	5	2	0
novel	270	16	13	2

**Figure 1:**
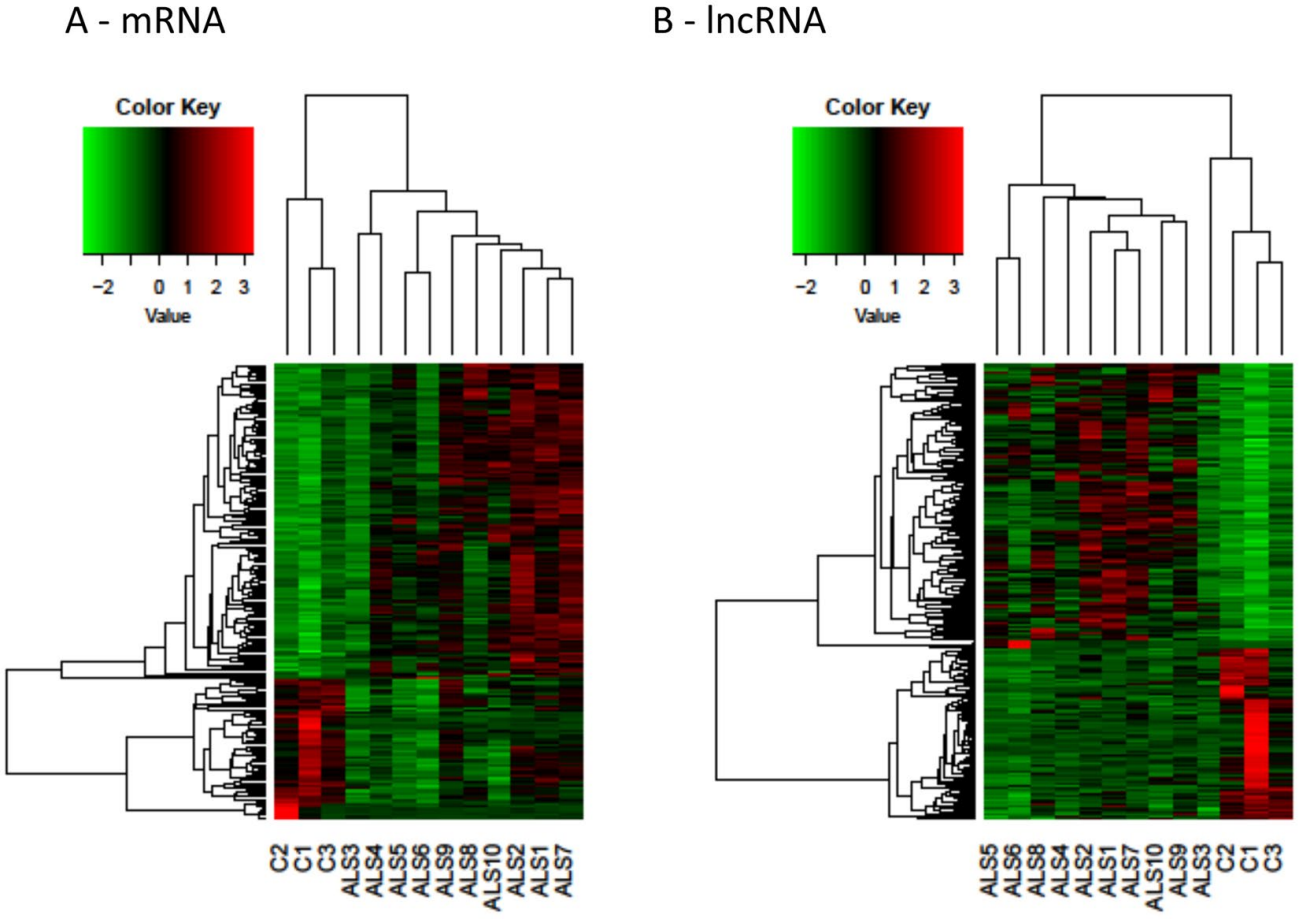
Expression profiles of differently expressed genes in ALS and healthy controls. In panel (A), differentially expressed mRNAs are shown, while in panel (B) differentially expressed lncRNAs are shown. All comparisons are given between the disease state and the control samples. We considered as differentially expressed only genes showing |log2(disease sample/healthy donor)| ≥ 1 and a False Discovery Rate ≤ 0.1.

Heat-maps of the differentially expressed mRNAs and lncRNAs in SALS relative to healthy controls are shown in Fig. 1A,B, respectively. Different expression profiles in SALS and healthy controls can be visibly distinguished. Considering the most deregulated lncRNAs in SALS group respect to healthy controls (Supplementary Table S1), it is evident that transcription pathway is highly involved. Three lncRNAs reported in the top 10 are described as antisense of transcription-related genes: *ZEB1-AS1* is indeed antisense of *ZEB1* transcription factor, *ZBTB11-AS1* is the antisense of *ZBTB11* gene, involved in the DNA binding and in transcriptional regulation, and also XXbac-BPG252P9.10 is described as the antisense transcript of IER3, involved in transcription. More in detail, it is known that IER3 is a transcription factor of the nuclear factor-kappa-B/rel (NF-kappa-B) family^23^, known to be involved in ALS^22^.

In *FUS* mutated patients, 21 deregulated long non-coding RNAs were identified. 11 of them were antisense, 6 were reported as lincRNAs while the remaining were categorized as processed transcripts (2) or sense intronic RNAs (2) (Table 1, Table 2, Supplementary Table S2). Five out of the 21 deregulated lncRNAs were reported as “known”.

For *TARDBP* mutated patients, 15 DE LncRNAs were detected: 7 antisense, of which only one was already described, 6 lincRNAs, 1 processed transcript and 1 sense intron RNA (Table 1, Table 2, Supplementary Table S3).

In *SOD1* mutated patients, we detected only 2 deregulated lncRNAs, both reported as novel antisense RNAs. (Table 1, Table 2, Supplementary Table S4).

### Validation of deregulated processed transcripts and antisense lncRNAs

To confirm RNA-seq results, we performed Real Time PCR (qPCR) for a subset of selected lncRNAs. The lncRNAs to be validated were selected based on these criteria: i) we preferably chose the transcripts to be validated among the 10 most differentially expressed lncRNAs found in each group; ii) we preferably analysed known antisense and processed transcripts; iii) we extended validation to other transcripts to confirm both up- and down-regulated genes iv) we included transcripts deregulated in all groups (Supplementary files 1 and Supplementary Tables S1, S2, S3 and S4).

We also investigated the RNA deregulation in spinal cord, derived from a post-mortem explant in sporadic ALS patients, which is a tissue known to be involved in ALS^24^.

#### SALS patients

**Figure 2:**
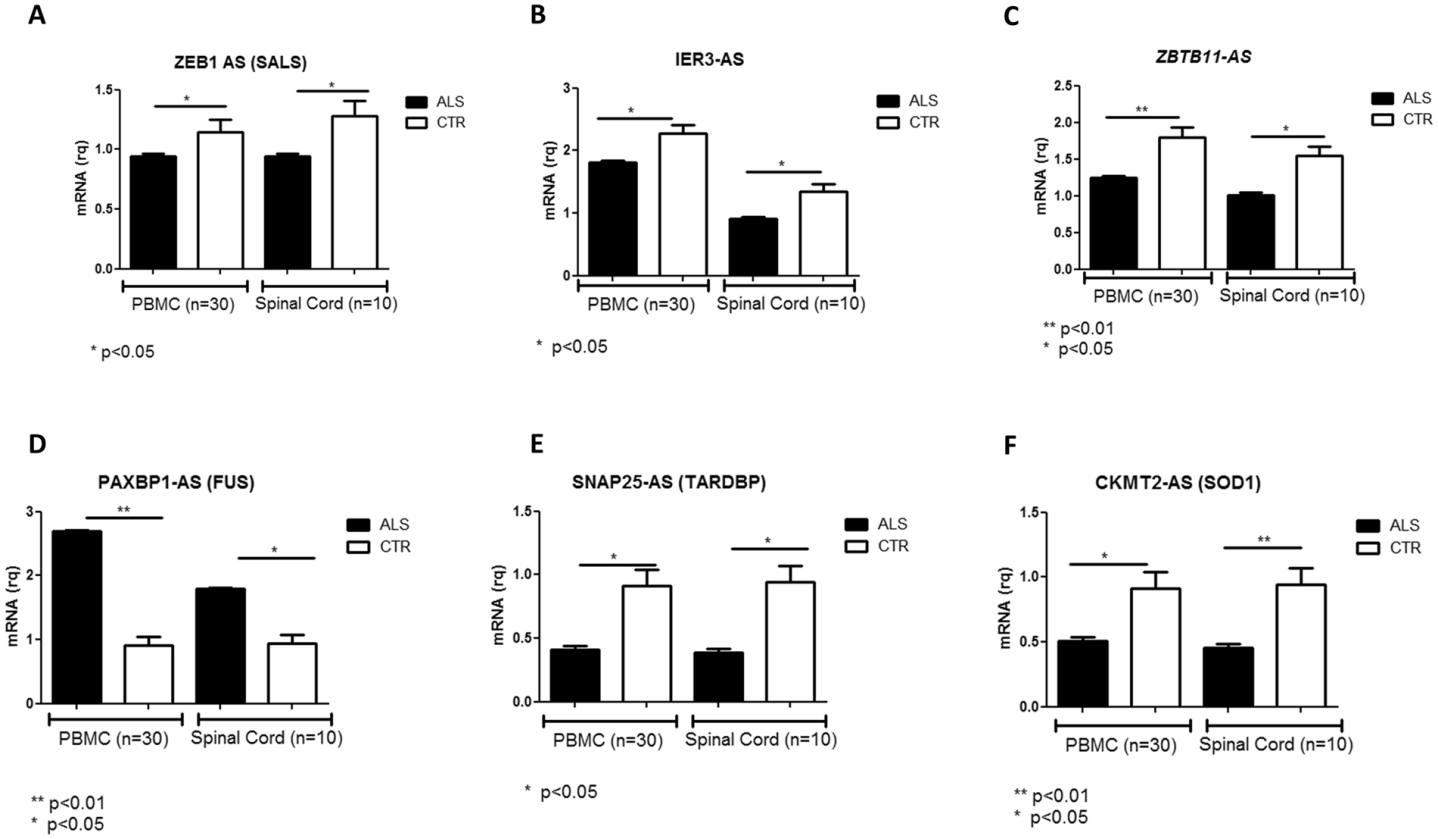
Differentially expressed transcripts verified by Real Time PCR in PBMC and Spinal Cord from larger cohort of SALS and CTRs. (**A**) ENST00000423714.1 (ZEB1-AS1); (**B**) ENST00000607333.1 (IER3-AS); (**C**) ENST00000536865.1 (ZBTB11-AS); (**D**) ENST00000458479.1 (PAXBP-AS); (**C**) ENST00000438646.1 (SNAP25-AS); (**D**) ENST00000502041.2 (CKMT2-AS). In brackets in panel titles, the group in which the transcript was found deregulated is reported.

In SALS patients, we validated antisense lncRNAs and processed transcripts with deregulation ≥ 1 in terms of |Log2FC| (ZEB1-AS1, XXbac-BPG252P9.10 IER3-AS, ZBTB11-AS1, RP11-475I24.8, RP11-38M8.1 and ENST00000417346*)*. Even if TTC25 and SPON1 were categorized as lncRNAs in the manually annotated GENCODE catalogue, both were described as well-known coding RNAs in RefSeq and ENSEMBL. Thus, they were no object of RT validation. Results are represented in Fig. 2, panels A, B and C.

ENST00000423714.1 (*ZEB1-AS1*) was the first differentially expressed AS in SALS group and it was found down-regulated compared to controls (log_2_FC = −2.4). It was annotated as a processed transcript, although it is the known AS of Zinc Finger E-Box Binding Homeobox 1 gene *(ZEB1*), acting as a transcriptional repressor by chromatin and E-box binding (Fig. 2A). ENST00000607333.1 *(XXbac-BPG252P9.10)* was annotated as AS of IER3, one of the transcription factors of (NF-kappa-B) family, with a crucial role in cell survival by regulation of anti-apoptotic genes. In SALS patients, XXbac-BPG252P9.10 was down regulated (log_2_FC = −2.35) compared to healthy subjects (Fig. 2B). ENST00000536865.1 (*ZBTB11-AS1*) was found down-regulated (log_2_FC = −2.14) in SALS patients compared to controls both in PBMC and Spinal cord (Fig. 2C). It was annotated as antisense of Zinc Finger and BTB Domain Containing 11 gene (ZBTB11), involved in DNA binding and in transcriptional regulation.

#### FUS mutated patients

ENST00000458479.1 (*PAXBP-AS*) was the only known differentially expressed AS in FUS patients. PAX3 and PAX7 Binding Protein AS was up-regulated (log_2_FC = 2.69) in FUS patients (Fig. 2D). GO annotations related to this gene included transcription factor activity, sequence-specific DNA binding and RNA polymerase II core promoter proximal region sequence-specific DNA binding.

#### TARDBP mutated patients

ENST00000438646.1 (*SNAP25-AS*), in the top 10 of differentially expressed lncRNAs, was the first AS found deregulated (down regulated, log_2_FC = −2.34) in TARDBP group of patients (Fig. 2E). This gene is involved in axonal repair and synaptic vesicle processing and it was previously reported to be deregulated in ALS patients^25^.

#### SOD1 mutated patients

ENST00000502041.2 (*CKMT2-AS*) was the only lncRNA deregulated (down-regulated, log2FC = −2.08) in SOD1 mutated patients (Fig. 2F). It is annotated as CKMT2 antisense RNA, which is a Mitochondrial creatine kinase (MtCK).

### Pathway analysis of lncRNAs

Non-coding RNAs pathway analysis was performed using LncPath R package, where a pre-computed lncRNA-mRNA relationship network was used to evaluate the extent of each gene influenced by DE lncRNAs (https://CRAN.R-project.org/package=LncPath). We detected some pathways synergistically regulated by lncRNA sets (Table 3). Most interesting pathways referred to Mapk signalling, cytokine receptor interaction, chemokine signalling, natural killer cell mediated cytotoxicity and regulation of actin cytoskeleton.

**Table 3:** Non-coding RNAs pathway analysis results. LncRNAs pathway analysis was performed to explore pathways significantly enriched by genes influenced by DE lncRNAs. Pathway name, gene set size (i.e.: the number of genes in each specific pathway affected by DE lncRNAs) and p-values are reported.

Gene Set Name	Gene Set Size	P Value
KEGG_SPLICEOSOME	78	0
KEGG_MAPK_SIGNALING_PATHWAY	76	0
KEGG_ERBB_SIGNALING_PATHWAY	51	0
KEGG_CALCIUM_SIGNALING_PATHWAY	24	0
KEGG_CYTOKINE_CYTOKINE_RECEPTOR_INTERACTION	25	0
KEGG_CHEMOKINE_SIGNALING_PATHWAY	42	0
KEGG_ENDOCYTOSIS	38	0
KEGG_FOCAL_ADHESION	48	0
KEGG_JAK_STAT_SIGNALING_PATHWAY	30	0
KEGG_NATURAL_KILLER_CELL_MEDIATED_CYTOTOXICITY	27	0
KEGG_LONG_TERM_POTENTIATION	19	0
KEGG_NEUROTROPHIN_SIGNALING_PATHWAY	47	0
KEGG_REGULATION_OF_ACTIN_CYTOSKELETON	54	0
KEGG_INSULIN_SIGNALING_PATHWAY	31	0
KEGG_MELANOGENESIS	20	0
KEGG_PATHWAYS_IN_CANCER	97	0
KEGG_GLIOMA	33	0
KEGG_PROSTATE_CANCER	32	0
KEGG_MELANOMA	38	0
KEGG_SYSTEMIC_LUPUS_ERYTHEMATOSUS	36	0
KEGG_FC_GAMMA_R_MEDIATED_PHAGOCYTOSIS	19	0,001
KEGG_CHRONIC_MYELOID_LEUKEMIA	32	0,001
KEGG_VEGF_SIGNALING_PATHWAY	19	0,002
KEGG_ENDOMETRIAL_CANCER	18	0,002

### Deep sequencing mRNAs expression profiles

We performed RNA-seq analyses also to determinate the expression profiles of mRNAs of ALS patients compared to matched healthy subjects. In SALS population, RNA-seq data showed 87 differentially expressed mRNAs, 30 of which down-regulated while 57 up-regulated (Table 1, Supplementary Table S5). Heat-map representing the expression levels of all dysregulated mRNAs in SALS and healthy subjects is represented in Fig. 1A.

In mutated ALS, PBMCs clearly showed different mRNA profiles between patient groups. We detected 122 altered genes in FUS group, 30 in TARDBP and 18 in SOD1 patients (Table 1 and Supplementary Tables S6, S7 and S8).

mRNAs sequence data analysis showed a common profile between TARDBP and FUS groups and between SALS and SOD1 groups. In fact, patients mutated in TARDBP showed a 67% of down-regulated and 33% of up-regulated mRNAs, and FUS patients showed a similar regulation, 71% of down-regulated genes and 29% of up-regulated genes. On the other hand, SOD1 and SALS groups showed a similar profiling with a major number of up-regulated genes (78% and 66% respectively). Only one gene, Two-pore channel 1 (*TPCN1*), has been found in common between the different groups. Interestingly, this gene is involved in Mapk signaling pathway, already identified by our lncRNAs pathway analysis (Table 3).

### mRNA pathway analysis

**Figure 3:**
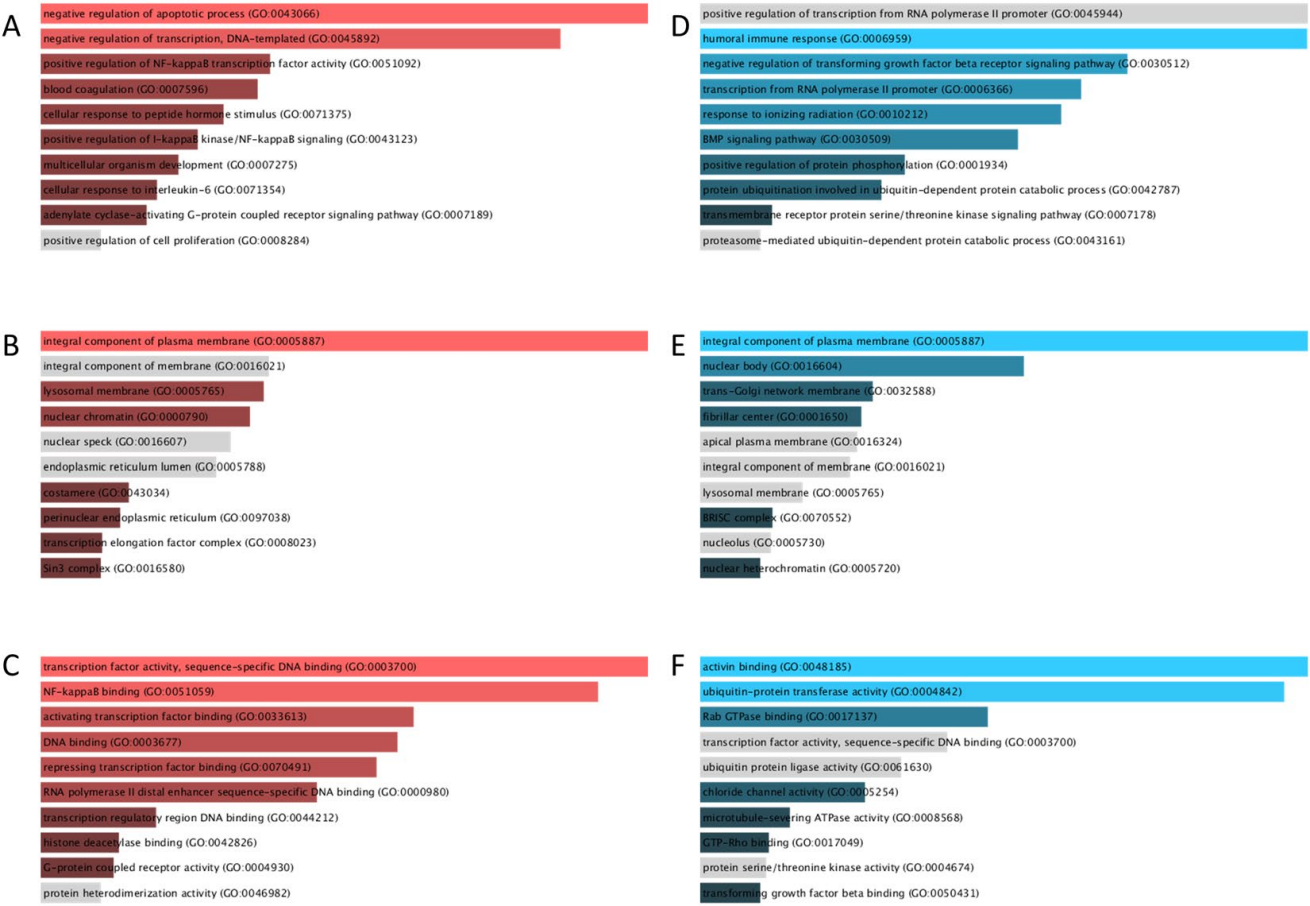
GO analysis for DE genes in SALS patients compared to healthy controls. TOP10 enriched GO terms for Biological process (**A**,**D**), cellular component (**B**,**E**) and molecular function (**C**,**F**). The length of the bar represents the significance of that specific gene-set or term. The brighter the color, the more significant that term is. Panels (A,B) and (C) have been obtained considering down-regulated genes, while panel (D,E) and (F) considering up-regulated genes.

**Figure 4:**
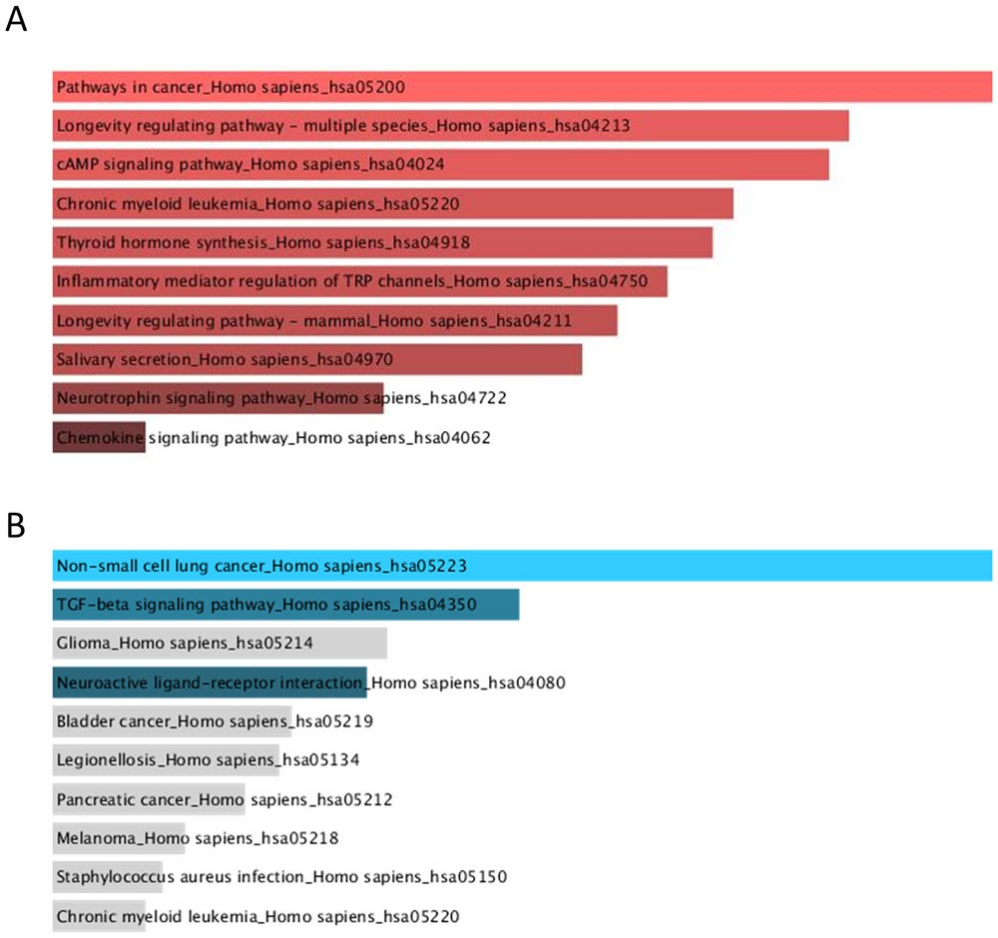
KEGG pathway analysis for DE genes in SALS patients compared to healthy controls. TOP10 KEGG pathways enriched by deregulated genes are shown. The length of the bar represents the significance of that specific gene-set or term. The brighter the color, the more significant that term is. Panels A has been obtained considering down-regulated genes, while panel B considering up-regulated genes.

GO terms enrichment and KEGG pathway analysis for DEGs in SALS patients compared to healthy controls has been performed for up-regulated and down-regulated DEGs, separately^26^ (Fig. 3, Fig. 4).

The GO biological processes enriched terms for down-regulated genes are related to apoptotic process and transcription regulation (Fig. 3A). Up-regulated genes affect humoral immune response and negative regulation of transforming growth factor beta-receptor signalling pathway (Fig. 3D). Enriched GO terms for Cellular Component include Integral Component of plasma membrane both for up- and down-regulated mRNAs (Fig. 3B,E). With respect to molecular function, the most enriched GO terms targeted by down-regulated mRNAs include transcription factor activity, NF-kappaB binding, activating transcript factor binding and DNA binding (Fig. 3C). The highest enriched GO terms targeted by up-regulated transcripts included activin binding and ubiquitin-protein transferase activity (Fig. 3F). KEGG pathways enriched by dysregulated genes include also cancer-related pathways both for up-and down-regulated mRNAs (Fig. 4A,B).

### Coding/non-coding co-expression analysis

Coding non-coding RNAs co-expression network was constructed via WGNCA R package and drawn using Cytoscape software (http://www.cytoscape.org/).

**Figure 5:**
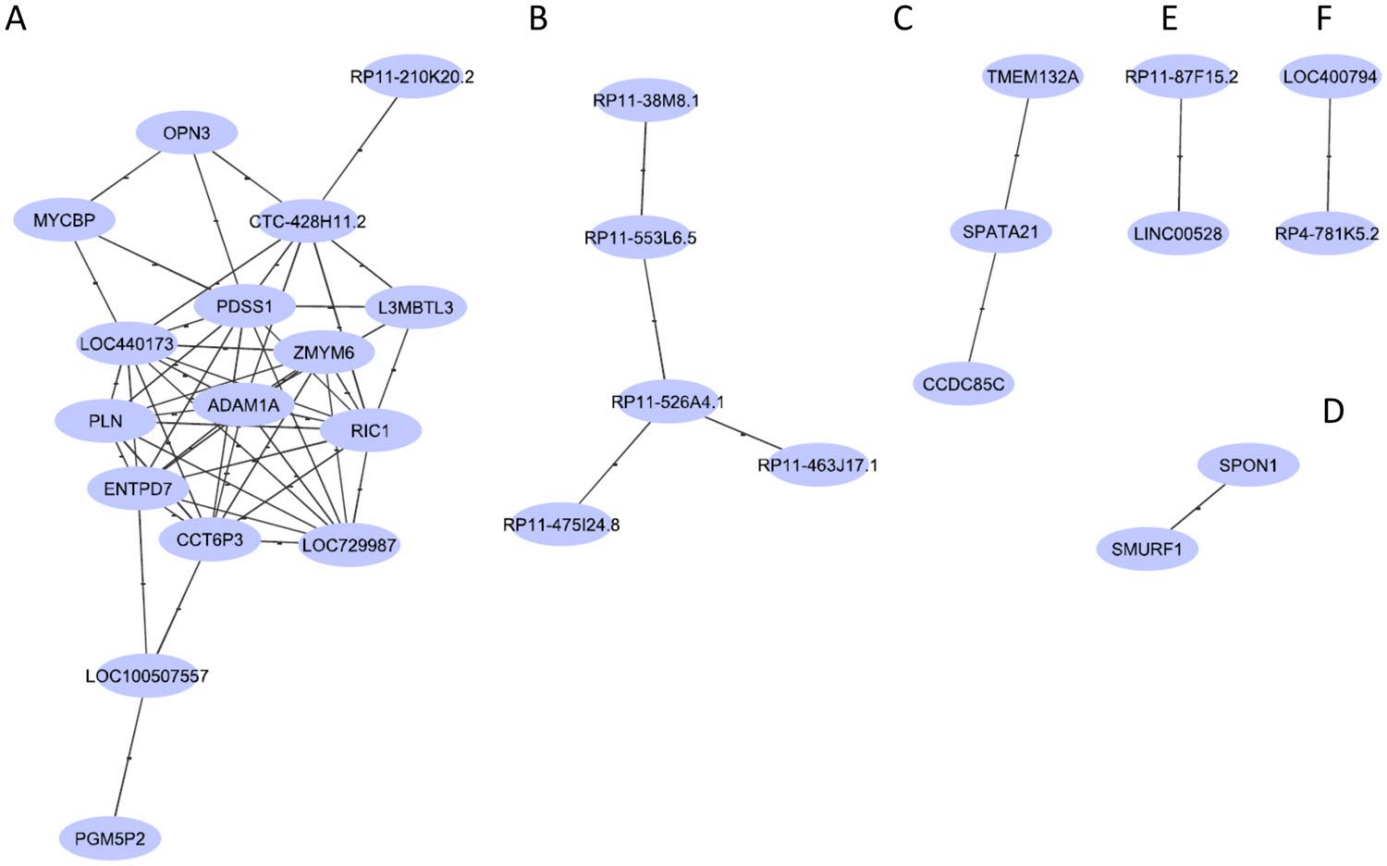
Coding non-coding RNAs co-expression analysis. The network represents co-expression correlations between mRNAs and lncRNAs. Six representative nets are shown. Correlation threshold was set to 0, 4.

Top 30 most deregulated coding and non-coding genes in SALS group respect to healthy controls were used to construct co-expression network. The three interesting subnets are shown in Fig. 5. Six clusters of co-expressed genes were observed. No antisense RNA was present in these networks. The first network involved 14 coding genes and 2 long intergenic RNAs (Fig. 5A). The second network contained lincRNAs only (Fig. 5B). Two clusters of coding genes only were than reported (Fig. 5C,D). Finally, two clusters containing lincRNAs only were represented (Fig. 5E,F).

## Discussion

The importance of various classes of regulatory non-coding RNAs (ncRNAs) in different diseases is increasingly being recognized. We performed a full profiling, by RNA-Seq approach, of the lncRNAs and mRNAs in human sporadic and mutated ALS patients, and healthy controls with the aim of extending our knowledge on molecular alterations of transcriptome and obtaining new data about its regulation.

Different classes of lncRNAs have been described^7^ but their characteristics are often unknown.

At first, we analysed the DE lncRNAs with a specific focus on Antisense RNA (AS). AS is a class of long non-coding RNAs that plays important regulatory roles in a variety of biological processes and they are required for proper regulation of coding genes^27^. AS lncRNAs may control the epigenetic state of chromatin, modulating methylation of DNA and/or histones or by removing repressors^27,28^. They promote sense gene transcription by recruiting transcription factors to enhance or modulate splicing of sense pre-mRNA^29,30^. Importantly, AS transcripts may regulate the half-life of their sense partners by establishing Dicer-dependent cutting of dsRNA, potentially followed by siRNA-mediated gene silencing^30^.

A characterization of AS has been developed in other neurodegenerative diseases, such as Parkinson’s Disease, showing an altered regulation of S/AS expression, and functional studies showed an important effect on neuron survival^31,32^. Our work is the first study concerning a deep screening on both coding and non-coding RNAs in ALS patients.

Considering SALS patient’s group, in the top 10 of DE lncRNAs, our data showed an interesting AS deregulation of genes involved in transcription regulation pathway such as *ZEB1-AS* and *ZBTB11-AS*. *ZEB1* may act as repressor or activator of transcription^33^. It may repress histones organization or activate chromatin regulators^33,34^. Moreover, *ZEB1-AS* was studied in cancer, in fact it was demonstrated that higher expression values of *ZEB1-AS* promote tumor metastasis^35^. Another AS of a transcription factor, *ZBTB11-AS* was found decreased in SALS patients compared to controls (Table S1). It is annotated as AS of Zinc finger and BTB domain-containing protein 11 (*ZBTB11*) gene. It seems to be a negative regulator of cell cycle, even if it is not well characterized. It was partially studied in hepatocellular carcinoma and it was recently described as transcriptional repressor^36^.

Some of the sense genes regulated by the DE AS lncRNAs in SALS are already linked to neurodegenerative disease, such as *UBXN7-AS*^37^ *ATG10-AS*^38^ and *ADORA2A-AS*^39^. In fact, *UBXN7* is an ubiquitin protein bound by VCP, a known ALS protein. The regulation of *UBXN7* by its AS regulated the ubiquitination in ALS disease. *ATG10* was reported in the pathological pathway^37^ while *ADORA2A* is involved in neurodegenerative diseases as Huntington and Parkinson’s disorders in relation to defects in DNA methylation^31^. The role of DNA methylation is object of intensive studies in ALS but to date it has not been totally clarified^31^.

In *FUS* mutated patients, the most interesting data concern *PAX, a* fundamental for skeletal muscle development already described as involved in ALS^40^. In *TARDBP* group of patients *SNAP25-AS* was found DE. SNAP25 is involved in axonal repair and synaptic vesicle processing and it is deregulated in ALS patients^25,41^. In ALS mice model, human TDP-43 decrease the RNA levels of synaptic proteins (as SNAP25)^25,41^ and we cannot exclude that SNAP25-AS may be involved in this deregulation. Finally, SOD1 mutated patients showed only two DE lncRNAs, one of these annotated as *CKMT2* antisense. In ALS, a mutation in SOD1, which may lead to reduced creatine kinase activity by inactivation of important target enzymes, including MtCK, was described^42^.

In the second part of the paper we also analysed the mRNAs expression in both non-mutated and mutated patients.

Only one gene *(TPCN1*) was found in common between all ALS groups. This gene has an important role in autophagy pathway^43^ that is known to be altered in ALS^44,45^. TPCN1 is down-regulated in ALS patients compared to control, suggesting that TPCN1 reduction may act on autophagy functions.

As TPCN1, the top 10 of DEG mRNAs showed a general trend of down-regulation. The impact of these genes on the transcription pathway was confirmed by GO enrichment analysis (Fig. 3A): transcription regulation is indeed the second most involved pathway in SALS patients. About this pathway, *ZMYM6, TTF2* and *TAF5L* (Supplementary Table S5) are associated to nucleic acid binding and transcription^46^. This association emerges also respect to molecular function, The most enriched GO terms targeted by down-regulated mRNAs include transcription factor activity, activating transcript factor binding and DNA binding (Fig. 3C).

Furthermore, the apoptotic process is the first in the enriched GO terms for Biological process (Fig. 4A). Interestingly, about apoptosis, one of the deregulated gene detected in this work is NAIP, already described associate do ALS and SMN^47^ even if the role of apoptosis in ALS is still controversial^48,49^. Moreover, KEGG pathways include cancer-related pathways both for up-and down-regulated mRNAs (Fig. 3A,B). These data are potentially interesting because the two DE AS (ZEB1 and ZBTB11 AS) have been largely associated to cancer^33,35^. In addition, between the most DE genes MYCBP, the binding protein of MYC, is present. It is an important oncogene well characterized in cancer^50^ (Table S5). About KEGG analysis, it is worth noting the important involvement of the immune system pathways (Table 3). In fact, in the top 10 we have found i) cytokine-cytokine receptor interaction, ii) chemokine signalling pathway, iii) natural killer cell mediated cytotoxicity. We suppose that these data may be associated to the kind of samples (PBMC) that we used for RNA-seq analysis, moreover, the association between ALS and immune deregulation has been already reported^51^. This investigation has confirmed the importance of extending our knowledge on molecular alterations of transcriptome and the significance of the classes of regulatory long non-coding RNAs, especially antisense RNA, in ALS disease. Morever, in this work, we have investigated the possible involvement of lncRNAs only at the beginning of the disease, future studies will be focused on the correlation between the progression of the disease and the RNAs profile.

Our data brought the light on the importance of Sense and Antisense RNA regulation in central and peripheral system, offering numerous starting points for new investigations about pathogenic mechanism involved in ALS disease.

## Materials and Methods

### Study Subjects

30 SALS patients and 30 age- and sex-matched healthy controls (CTR) were recruited after obtaining written informed consent (Table 4). A subset of subjects (10 ALS and 3 CTR) was deep-squenced while all samples were included for Real Time PCR experiments. With this approach, deep-sequencing, although on a small number of samples, allowed to explore the whole transcriptome at the level of coding and non coding genes in ALS patients when compared to healthy controls and to select a subset of interesting transcripts, to be further studied and validated by Real Time PCR. ALS patients underwent clinical and neurologic examination at IRCCS National Neurological Institute “C. Mondino” (Pavia, Italy). All patients were diagnosed with ALS as defined by El Escorial criteria. All SALS patients were analysed to exclude any causative mutations in SOD1, TARDBP, FUS, C9orf72, ANG and VCP genes. The control subjects were recruited at the Transfusional Service and Centre of Transplantation Immunology, Foundation San Matteo, IRCCS (Pavia, Italy). Moreover, also a cohort of mutated ALS patients (2 FUS, 3 SOD1 and 2 TARDBP mutated) (Table 4) was analysed and compared to three healthy controls.

**Table 4:** Baseline characteristics of subjects recruited for this study.

	CTRs	SALS	FUS	SOD1	TARDBP
	(n = 30)	(n = 30)	(n = 2)	(n = 3)	(n = 2)
**Age (M ± SD)**	49 ± 10,3	66,6 ± 10,1	52,9 ± 4,9	52 ± 10,39	64,5 ± 20,5
**Sex**					
Males n (%)	48%	45%	50%	66,6%	0%
Females n (%)	52%	55%	50%	33,3%	100%
**Onset**		Spinal (100%)	Spinal (100%)	Spinal (100%)	Spinal (100%)
**ALSFRS**		41,15 [39,42–42,88]	41 [39,04–42,96]	39 [37,04–40,96]	42,5 [39,56–45,44]

Age values are reported as average ± standard deviation. The percentage of male and female subjects and the site of onset are also indicated. ALSFRS score is indicated as average and 95% confidence interval.

The study protocol to obtain PBMC from patients and controls was approved by the Ethical Committee of the National Neurological Institute “C. Mondino”, IRCCS (Pavia, Italy). Before being enrolled, the subjects participating in the study signed an informed consent form (Protocol n°375/04 – version 07/01/2004).

Spinal cord tissue was obtained from the Human Brain and Spinal Fluid Resource Center (VA West Los Angeles Healthcare center, Los Angeles, CA 90073), which is sponsored by NINDS/NIMH, National Multiple Sclerosis Society, and Department of Veteran Affairs. All experiments were performed in accordance with relevant guidelines and regulations.

### Isolation of human peripheral blood mononuclear cells (PBMC)

Peripheral blood mononuclear cells (PBMC) were prepared by centrifugation. Peripheral blood was layered (density = 1.077) and centrifuged at 950 g for 30 min. After isolation on a Ficoll-Histopaque layer (Sigma, Italy), cell viability was assayed by a trypan blue exclusion test and the cells were used for RNA extraction.

### RNA extraction

Samples were homogenized and total RNA was isolated by Trizol® reagent (Life Science Technologies, Italy) following the manufacturer’s specifications. RNAs were quantified using a Nanodrop ND-100 Spectrophotometer (Nanodrop Technologies, Wilmington, USA) and a 2100 Bioanalyzer (Agilent RNA 6000 Nano Kit, Waldbronn, Germany); RNAs with a 260:280 ratio of ≥1.5 and an RNA integrity number of ≥8 were subjected to deep sequencing.

### Libraries preparation for RNA-Seq and bioinformatic data analysis

Sequencing libraries were prepared with the Illumina TruSeq Stranded RNA Library Prep, version 2, Protocol D, using 500-ng total RNA (Illumina). Qualities of sequencing libraries were assessed by 2100 Bioanalyzer with a DNA1000 assay. Libraries were quantified by qPCR using the KAPA Library Quantification kit for Illumina sequencing platforms (KAPA Biosystems); RNA processing was carried out using Illumina NextSeq. 500 Sequencing. FastQ files were generated via llumina bcl2fastq2 (Version 2.17.1.14 - http://support.illumina.com/downloads/bcl2fastq-conversion-software-v217.html) starting from raw sequencing reads produced by Illumina NextSeq sequencer. Gene and transcript intensities were computed using STAR/RSEM software^52^ using Gencode Release 19 (GRCh37.p13) as a reference, using the “stranded” option. Differential expression analysis for mRNA was performed using R package EBSeq^53^. This tool was selected because of its superior performance in identifying isoforms differential expression^54^.

Differential expression analysis for long non-coding RNAs was performed with the R package DESeq. 2^55^. Coding and non coding genes were considered differentially expressed and retained for further analysis with |log_2_(disease sample/healthy control)| ≥ 1 and a FDR ≤ 0.1. We imposed minimum |Log_2_FC| of 1 and a FDR lower than 0.1 as thresholds to differentially expressed genes. This choice is motivated by the decision to maximize the sensitivity of this analysis, in order to perform a massive screening and identify candidate genes to be validated with a wider sample population with real-time analysis.

### Pathway and co-expression analysis

Gene enrichment analysis was performed on coding genes^26^. We performed a Gene Ontology (GO) analysis for biological processes, cellular components and molecular function and a Kegg pathway analysis (Kyoto Encyclopedia of Genes and Genomes http://www.genome.ad.jp/kegg) via enrichR web tool^56,57^.

Non-coding RNAs pathway analysis was performed. LncPath R package was used to map differentially expressed lncRNAs on a lncRNA-mRNA relationship network, to evaluate the extent of each gene influenced by lncRNAs, based on a network diffusion strategy(https://CRAN.R-project.org/package=LncPath).

Furthermore, a weighted gene co-expression network analysis was performed to assess functional annotation using WGCNA R package^58^. The 30 most deregulated coding and non-coding genes (in terms of log_2_FC) in SALS patients compared to healthy controls were selected for this analysis. Co-expression analysis of lncRNAs with well-annotated protein-coding genes can provide an approach to investigate the biological role of lncRNAs. Coding non-coding RNAs co-expression network was constructed via WGNCA R package^58^. Network nodes represent gene expression profiles, while undirected edges values are the pairwise correlations between gene expressions. Cytoscape software (http://www.cytoscape.org/) was used for network import and visualization.

### Real Time PCR

Using human gene sequences available from NCBI (www.ncbi.nlm.nih.gov/nucleotide), PCR oligonucleotide for sense genes pairs were selected spanning introns to optimize amplification from mRNA templates and avoiding nonspecific amplification products, using NCBI’s Primer- BLAST or online Primer 3.0. Moreover, primers were designed in specific regions that do not overlap with Antisense sequences (primers upon request). Total cDNAs were prepared from 1 to 2 μg of total RNA using SuperScript III reverse transcriptase (LifeTechnologies, SanDiego, CA). qPCR reactions included 200 nM of each oligonucleotide, 1 ul of SYBR Green SuperMix (BioRad, Richmond, CA), and 1 μL of cDNA template (or water control). Cycling conditions using a BioRadiQ5 Real-Time thermocycler were 5 min denaturing at 95 °C, followed by 40 cycles of 95 °C (10 s) and 58 °C annealing (30 s).

### Real Time Data Analysis

Cycle threshold (Ct) values were automatically recorded for each replicate qPCR reaction, and mean Ct values were normalized against those determined for GAPDH. Fold-expression differences relative to healthy controls were determined using the 2ΔΔCt method. Significance of gene expression changes relative to controls was analysed using one-way ANOVA (Kruskal-Wallis) and the Dunns post-test for all possible test pairings using Prism GraphPad 3.03 software (GraphPad Software, San Diego, CA). P-values (two tailed) with 95% confidence intervals were computed, and P < 0.05 was considered statistically significant.

### Data availability

The sequencing data obtained in this study were deposited in NCBI GEO [GSE106443].

## Electronic supplementary material

Supplementary information

Supplementary tables 1
