# Supplementary material for: Long non-coding and coding RNAs characterization in Peripheral Blood Mononuclear Cells and Spinal Cord from Amyotrophic Lateral Sclerosis patients
Source: Sci Rep. 2018 Feb 5;8:2378. doi: 10.1038/s41598-018-20679-5 (PMC5799454; doi:10.1038/s41598-018-20679-5)

**Real Time validation for coding and long non coding transcripts**

To validate RNA-seq results, we performed Real Time PCR (qPCR) on the same RNAs samples that we used for libraries preparation (primers available upon request). We have select a number of candidates (both mRNAs and lncRNAs), among up- and down-regulated in each group, and we have performed the validation in SALS (N=10), FUS (N=3), SOD1 (N=2) and TARDBP (N=2) samples, respectively.

Unfortunately, only 2 samples instead than 3 mutated in SOD1 gene were available for all the experiments. About TARDBP mutated patients, the few amount of RNAs available after this work allowed us to validate only one mRNAs and 4 lncRNAs.

About SALS patients, we have analysed by Real Time PCR 5 mRNAs (LOC729987, KIAA2013, HDAC1, MYCBP and TPCN1) and 6 lncRNAs. In fact, we add to ENST00000423714.1, ENST00000607333.1 and ENST00000536865.1 (already described in the paper), more 3 lncRNAs (ENST00000425493.1, ENST00000609619.1 and ENST00000417346.1) (Supplementary Figures 1 and 4).

About FUS, more 4 lncRNAs (to add to ENST00000458479.1), such as, ENST00000572565.1, ENST00000377540.1, ENST00000424587.2 and ENST00000417346.1 have been analysed and five mRNAs (FAM90A7P, DNM1P41, LOC148413, OR7E12P and TPCN1) have been run by Real Time PCR (Supplementary Figures 2 and 4).

As concern SOD1 mutated patients, the mRNAs validated were CCSAP, STK17A, BRAT1, TAF5L and TPCN1 while, about lncRNAs, only two (ENST00000502041.2 already reported in the manuscript, and ENST00000491934.2) have been found deregulated and we have run the validation by Real Time PCR (Supplementary Figures 3 and 4).

About TARDBP mutated patients, we have validated TPCN1 gene and 2 lncRNAs, such as ENST00000438646.1 (described in the paper) and ENST00000417346.1 (Supplementary Figure 4).

The validated lncRNAs were selected based on two criteria: i) we chose the 5 most differentially expressed lncRNAs found in each group; ii) we preferably analysed known antisense and processed transcripts.

Supplementary Figure 1 – Differentially expressed transcripts verified by Real Time PCR in PBMC in SALS group compared to healthy controls. Two non coding RNAs (A and B) and four coding RNAs (C, D, E and F) were assayed.


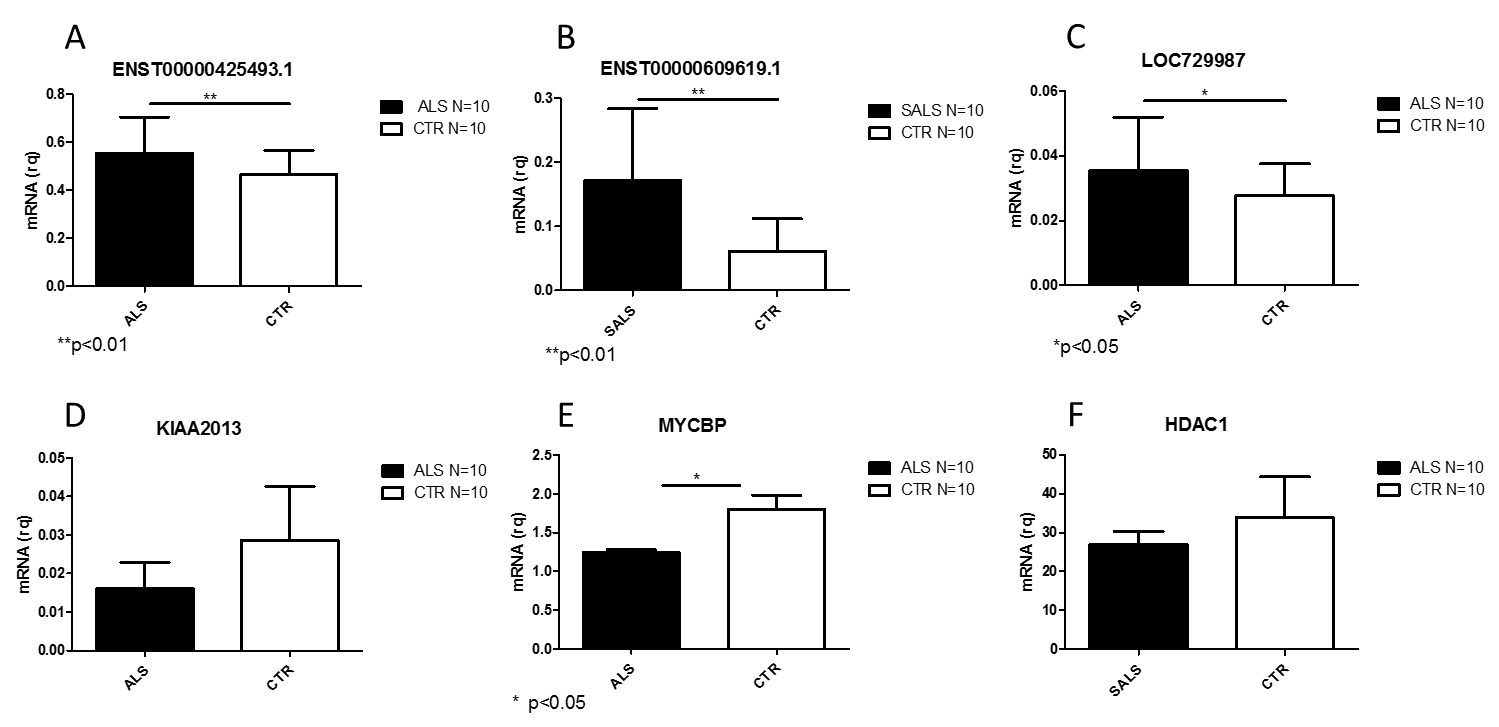


Supplementary Figure 2– Differentially expressed transcripts verified by Real Time PCR in PBMC in FUS group compared to healthy controls. Three non coding RNAs (A, B and C) and four coding RNAs (D, E, F and G) were assayed.


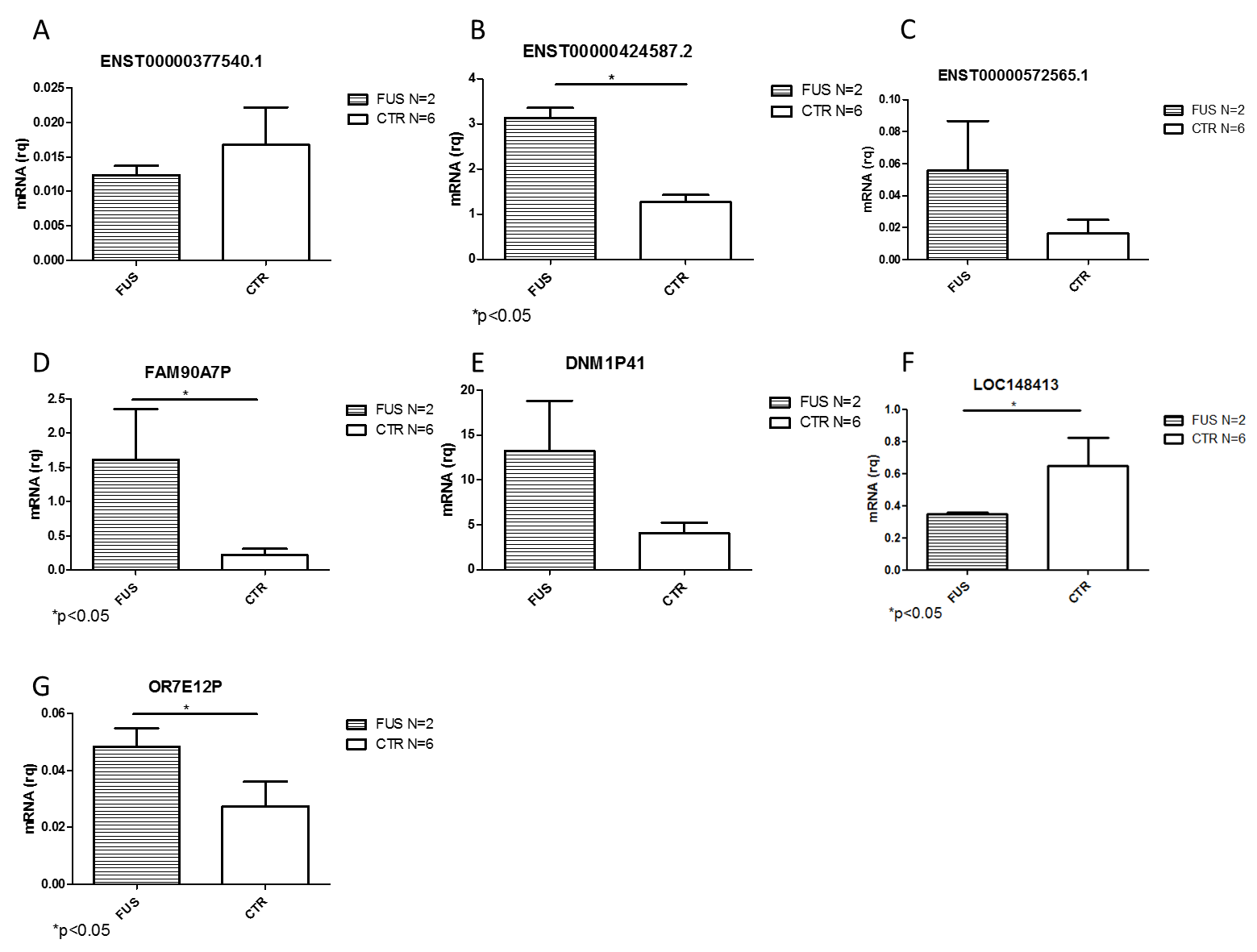


Supplementary Figure 3– Differentially expressed transcripts verified by Real Time PCR in PBMC in SOD1 group compared to healthy controls. One non coding RNA (A) and four coding RNAs (B, C, D and E) were assayed.


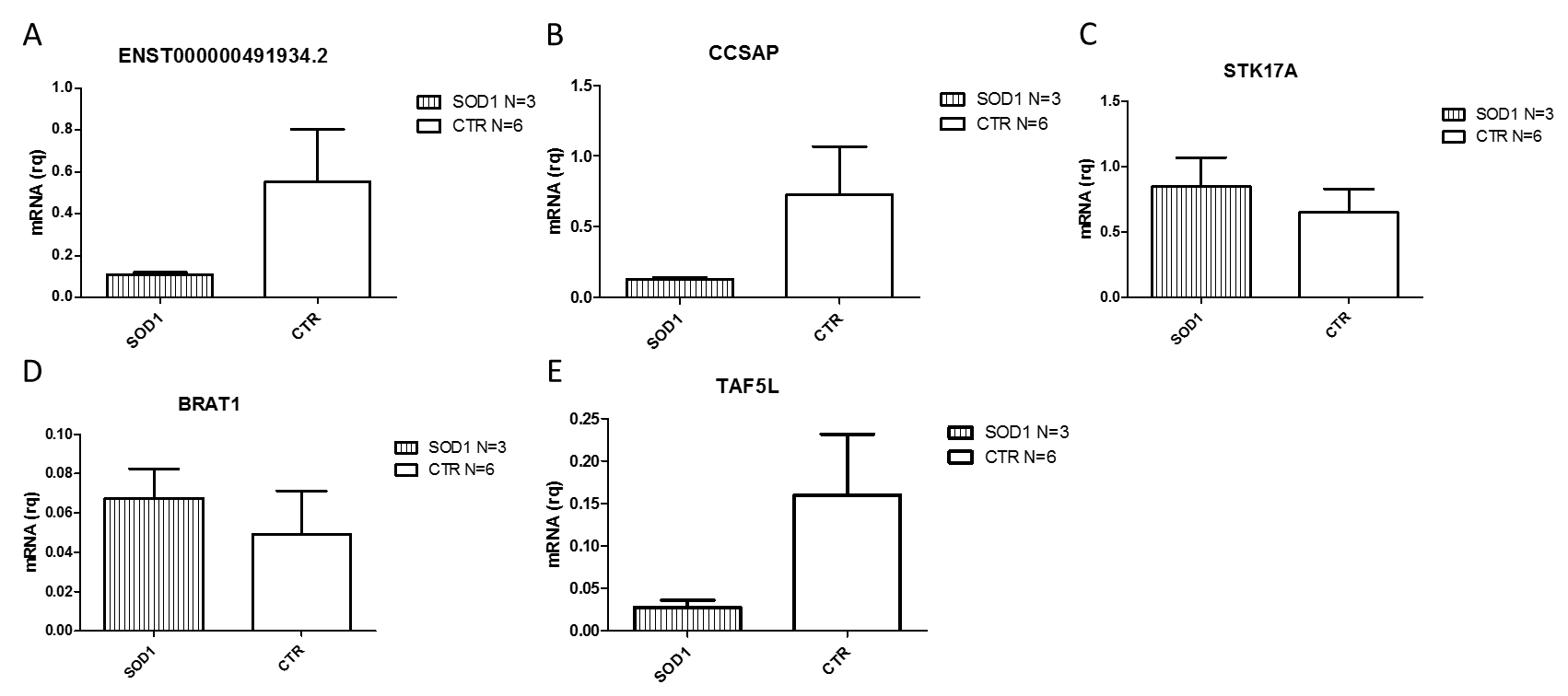


Supplementary figure 4 – Common differentially expressed transcripts verified by Real Time PCR in PBMC in all groups compared to healthy controls. One non coding RNA (A) and one coding RNA (B) were assayed. LncRNA ENST00000417346.1 was common to all groups with the exception of SOD1 mutated group (here, onle two DE lncRNAs were detected).


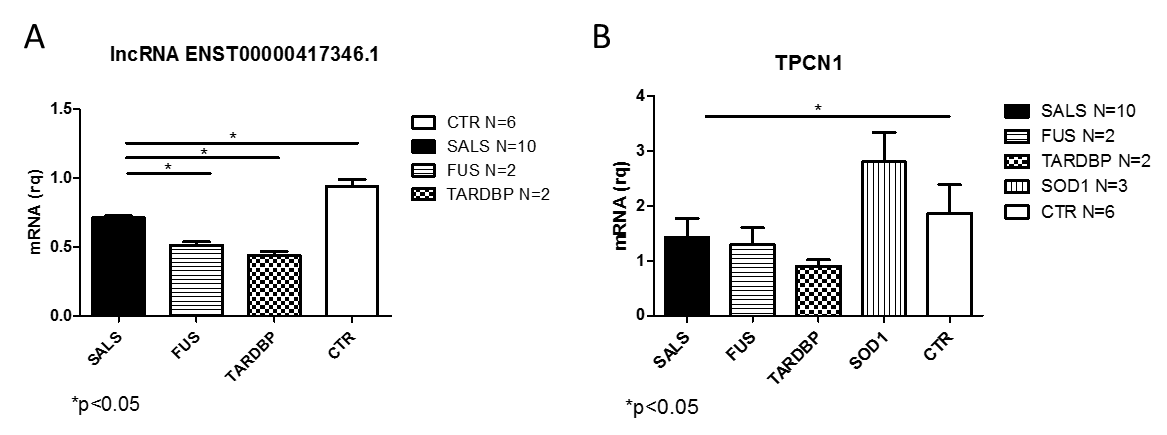

Supplement: Supplementary file 1 — Supplementary information [file 41598_2018_20679_MOESM1_ESM.docx]
